# The Time Course of Activation of Object Shape and Shape+Colour Representations during Memory Retrieval

**DOI:** 10.1371/journal.pone.0048550

**Published:** 2012-11-14

**Authors:** Toby J. Lloyd-Jones, Mark V. Roberts, E. Charles Leek, Nathalie C. Fouquet, Ewa G. Truchanowicz

**Affiliations:** 1 Swansea University, Wales, United Kingdom; 2 Bangor University, Wales, United Kingdom; 3 University of Birmingham, England, United Kingdom; 4 Wales Institute of Cognitive Neuroscience, Wales, United Kingdom; Baycrest Hospital, Canada

## Abstract

Little is known about the timing of activating memory for objects and their associated perceptual properties, such as colour, and yet this is important for theories of human cognition. We investigated the time course associated with early cognitive processes related to the activation of object shape and object shape+colour representations respectively, during memory retrieval as assessed by repetition priming in an event-related potential (ERP) study. The main findings were as follows: (1) we identified a unique early modulation of mean ERP amplitude during the N1 that was associated with the activation of object shape independently of colour; (2) we also found a subsequent early P2 modulation of mean amplitude over the same electrode clusters associated with the activation of object shape+colour representations; (3) these findings were apparent across both familiar (i.e., correctly coloured – *yellow banana*) and novel (i.e., incorrectly coloured - *blue strawberry*) objects; and (4) neither of the modulations of mean ERP amplitude were evident during the P3. Together the findings delineate the timing of object shape and colour memory systems and support the notion that perceptual representations of object shape mediate the retrieval of temporary shape+colour representations for familiar and novel objects.

## Introduction

A key debate in cognitive neuroscience concerns the extent to which the mind and brain is modular in organization [1], [2] and there is a growing body of evidence supporting the distinct representation of object properties including shape, colour and texture [3], [4]. However, such independence gives rise to a binding problem: when, where and how are these different object properties unified in a single object? This question has received much attention in the domain of visual perception and some attention recently in the domain of explicit memory (i.e., the conscious recollection of an event; [5]). In contrast, little is known about the binding of different object properties during implicit or unconscious retrieval as assessed by repetition priming (i.e., the benefit in processing a stimulus due to a repeated encounter with that stimulus) and yet this remains fundamental to models of human cognition. In particular, we investigated the timing of activating implicit memory for object shape and colour which is important for theory but largely unknown.

How are object properties bound together during visual perception? Based primarily on experiments in which subjects search for a target defined either by a simple feature or a conjunction of features, the highly influential feature integration theory (FIT [6]) proposes that visual features such as colour, curvature and orientation are processed in parallel in separate feature maps and later integrated through spatial attention or top-down processing. Supporting evidence comes from the *object reviewing paradigm*, in which subjects respond to letters or other objects that are preceded by task-irrelevant prime displays [7] (for a review, see [8]). For instance, performance is enhanced when the same letter appears in the prime and probe display and if prime and probe appear in the same location, and this reflects the binding of identity and location and the creation of an *object file*: repeating the same combination of identity and location allows for use of the previously created binding which benefits performance. A recent neuroimaging study provides support for the retrieval of object files. Keizer et al. [9], presented preview and probe displays consisting of two pictures showing a face and a house. Either the face or the house moved in one of two possible directions and participants were asked to respond to the direction of the probe display irrespective of which object moved. They found that when the probe display was the image of a face moving in a particular direction and the prime was an image of a house moving in the same direction, increases in activation of both the fusiform face area (coding face information) and also the right parahippocampal place area (coding house information) were observed. This suggests that feature binding creates episodic representations that tend to be reactivated as a whole as soon as one of the components matches the current input. Similar interactions between repetition effects and feature binding have been observed for other features including shape, colour and location [10], [11]. Additional support for FIT also comes from studies of binding auditory features [12], [13]. Note that, if the experimental design is such that there is no systematic benefit in the retention of stimulus (or response) information across trials, then the data most likely reflect inter-trial effects which result predominantly from automatic perceptual priming or dimensional weighting (i.e., one dimension such as colour may receive more attentional weight at the expense of other dimensions) rather than top-down control [14], [15]. Finally, a recent alternative to FIT proposes that there may be multiple dissociable binding processes, rather than a unitary binding mechanism, which are dependent on the nature of the feature dimension and have a specific neuroanatomical basis [16], [17]. For instance, it appears that there are neural substrates specifically related to conjunction search as compared with feature search, and specifically for cross-dimension shape-colour conjunctions (e.g., searching for an orange vertical bar among blue vertical bars and orange tilted bars) as compared to within-dimension shape conjunctions (e.g., searching for an upright T among non-target oriented Ts and Ls [18].

A different approach to understanding binding in visual perception has focused on the time course of encoding object features and conjunctions in object detection and identification tasks [19], [20]. It is clear that object features such as shape, colour and texture can be encoded very rapidly. For instance, Martinovic et al., [21] manipulated the amount and kind of object features (i.e., visual complexity, surface detail and object colour typicality) in a picture naming task with common objects, whilst measuring event-related potentials (ERPs). They found that early synchronizations (around 100 ms) increased when more features had to be encoded and later activity around 200–400 ms was modulated by the representation of object features encoding edges or colour. More recently, in an ERP study by Lu et al., [22], both early and later effects of surface colour were observed during object identification: ERP waves indexing early perceptual processes, including N1, P2 and N2, differentiated between objects in their appropriate colour (e.g., yellow banana) and those in an inappropriate colour (e.g., purple banana). This colour effect also occurred in N3, which has been associated with semantic processing of pictures but not in N4 which Lu et al suggest is associated with domain-general semantic processing [23].

In contrast, how might object properties be bound together during implicit memory retrieval? Implicit memory is usually assessed via *repetition priming*, whereby responses to previously encountered items are faster and less error prone than those to new items. Repetition priming is contingent upon the overlap of perceptual, conceptual and response-related processing during encoding and retrieval [24], [25]. For instance, behavioural priming is reduced when an item is presented in a different modality or format from study to test [26], [27]. Nevertheless, object priming is not normally influenced by study-to-test changes in features that do not contribute strongly to identification, such as size or left-right orientation, except when those features are relevant to the task at hand [28]. For instance, Srinivas [29] found that priming was influenced by the size of the object when both study and test tasks required a judgement about the real size of objects. This contrasts with episodic recognition where study-to-test changes more consistently influence memorial performance [5].

Importantly, whilst behavioural performance improves after a repeated encounter with an object, neocortical activity in humans tends to decrease - a phenomenon known as *repetition suppression* (for a recent review and commentaries, see [30]). A number of methods have been used to measure repetition suppression, including electrophysiology and fMRI, and it has been observed in multiple neocortical regions [31]. One prominent explanation concerns *sharpening*, whereby stimulus repetition strengthens or sharpens the representation of particular stimulus properties leading to a particular pattern of behavioural priming accompanied by long-term changes in cortical activity [32], [33]. As an object is presented repeatedly, neurons coding attributes that are not essential for identifying the object decrease their response. The result is that the representation becomes more selective, yielding enhanced behavioural performance. For instance, when a memory task involves retrieving only shape, neurons coding colour may decrease their responses and in doing so weaken their connections with shape neurons. This results in a predominantly shape-based representation mediating priming. In contrast, when the task involves retrieving both shape and colour knowledge there is no decrease in the responses of neurons coding colour relative to neurons coding shape resulting in a representation mediating priming which is both shape- and colour-based. This notion is predicated on the assumption that object shape and colour information is linked together in a neuronal network however the existing evidence on the relationship between these two kinds of information is equivocal [34], [35], [36]. Moreover, there are alternative theoretical possibilities. In brief, repetition suppression may reflect a more rapid overall time course of neural activity (e.g. [37], and a more sophisticated version of this account [38]) or enhanced neural synchronization whereby cells fire at lower overall rates and also more synchronously with each other, leading to more efficient neural processing [39].

We used ERPs to investigate the timing of activation of object shape and colour memory during implicit retrieval. We adopted a basic study-test priming procedure with a colour typicality rating task at study and a coloured-object decision task at test (i.e., making a speeded *yes* or *no* decision as to whether an object is correctly or incorrectly coloured). The stimuli were either *familiar* (i.e., correctly coloured – *yellow banana*) or *novel* (i.e., incorrectly coloured - *blue strawberry*) combinations of object shape and colour. Manipulations of object colour were carried out across study-test phases so that at test participants were presented with an object that remained in the same colour as at study (*same*), an object that changed its colour from study to test (*change*), or an unstudied object (*new*) that served as a baseline against which to measure priming. (Note, in this way changes in colour alone occurred during the experiment rather than combined changes in both shape and colour.) The logic was that the comparison of *new* and *change* conditions indexed the retrieval of memory for object shape as only shape was primed from study to test, and the comparison of *same* and *change* conditions indexed the retrieval of memory for object shape+colour representations as in the same condition both shape and colour were primed from study to test. In this way, we could elucidate the time course associated with the retrieval of shape and shape+colour representations, respectively.

The main question was whether the earliest sensitivity to the retrieval of shape+colour representations could be distinguished from the retrieval of shape representations. If it could, then we would have provided new evidence on the nature of the functional dissociation between object shape and colour as well as on the time course of retrieval of these different kinds of object information. In an ERP study by Proverbio et al. [40], participants detected a target by attending either to its shape or colour. Of particular interest, they found that when colour was the attended feature (e.g., *grey*) the N2 wave was greater when the colour was associatively related to the object (e.g., *elephant*) as compared to when it was not (e.g., *strawberry*). However, the reverse was not observed when shape was the attended feature. Thus, the selection of colour depended on object shape even when shape was not relevant to the task. We suggest that object shape may provide the *gateway* to associated perceptual object features such as colour, texture and size. Indeed, consistent with the primacy of object shape, priming for structurally plausible 3D objects has been taken to be the hallmark of the structural representation system proposed by Cooper, Schacter and colleagues (for a review, see [41]). Furthermore, other recent structural description models of object recognition have proposed the existence of multi-dimensional object representations in which individual shape elements (e.g., surfaces) are uniquely bound to associated properties such as colour and texture at a different subsequent level of representation [42], [43]. Such models would predict a temporal dissociation between access to shape information and access to associated properties such as colour and texture. Additionally, access to shape is predicted to precede access to the associated properties to which it is bound.

The comparison of familiar versus novel objects also allowed us to examine a further issue concerning the nature of the representations mediating retrieval of object shape and colour in priming tasks. On the one hand, pre-existing stored shape representations could contribute to shape+colour priming, in which case we would expect to observe a difference in the time course of retrieval for familiar as compared with novel objects. Alternatively, priming for both familiar and novel objects may arise from the construction of new, temporary shape+colour representations. In this case, the time course of retrieval would be expected to be the same for both kinds of object. Consistent with the latter notion, priming of stimulus shape and colour may be considered a special case of implicit learning which can take place after just a single study trial and has been observed for novel stimuli [44], [45]. Related to this idea, Wang et al. [46], [47], have shown that learning to discriminate between novel three-dimensional shapes, in particular learning to associate different views of the same novel shape and also to identify differences between novel shapes having associated views, can occur early in the time course of processing during the N1 wave.

The high temporal resolution of ERPs is well-suited to addressing these issues as it allows us to differentiate early perceptual effects from later semantic and explicit recognition effects and also post-perceptual decision processes [48], [49]. We focused on early latency effects because perceptual priming is considered to be a relatively automatic and fast process occurring before semantic analysis and explicit recognition takes place and we were therefore most interested in the posterior P1, N1 and P2 ERP waves. Importantly, these waveforms have also been associated with various aspects of the perceptual processing of objects, including visual attention, global-local processing of hierarchical stimuli, visual categorization, and the encoding of object structure and colour during on-line identification [50], [51], [52]. Nevertheless, we also examined the P3 in order to provide a contrast for our interpretations and to see whether similar activity might occur later on in the time course.

Following the logic outlined earlier, our approach was first to identify, a priori, reliable sensitivity to shape repetition (measured by the difference between *new* and *change* conditions) in the ERP. This would serve as a temporal localiser contrast to define an index or temporal marker for shape-related repetition independent of colour. Once this was established, we determined ERP sensitivity to the repetition of shape+colour representations (measured by the difference between *same* and *change* conditions). We predicted perceptual priming of object shape and, subsequently, priming of object shape+colour early in the time course of processing. We also expected that these effects would be apparent for both familiar and novel objects reflecting the construction and retrieval of new, temporary shape+colour representations.

## Results

The mean correct response times (RTs), standard errors, and percentage correct for each condition in the coloured-object decision test are given in Table 1. A trial was scored an error if participants gave an incorrect response. Analysis of the behavioural data consisted of a two-way repeated measures ANOVA with transformation (new vs. change vs. same) and colour (correct vs. incorrect) as factors. For both behavioural and ERP analyses we report effect sizes estimated using partial eta-squared (ηp2), which overall according to generally accepted criteria were large (.01 = small; .06 = medium; .14 = large [53]). We report only significant results, alpha level .05.

**Table 1:** Mean response times (msec) and percentage correct (%) for the coloured-object decision test; values in brackets represent standard error.

Transformation	Colour	*Mean*	*%*
New	Correct	1098 (69)	95
	Incorrect	1142 (75)	87
Change	Correct	985 (45)	90
	Incorrect	1088 (77)	90
Same	Correct	929 (46)	95
	Incorrect	1056 (61)	85

For RTs, there was a main effect of transformation, F(2,54) = 14.64, p<.001, ηp2 = .35. Pairwise t-tests revealed a difference for new (1120 msec) versus change (1037 msec) conditions, t(27) = −3.53, p = .002, demonstrating repetition priming for object shape, and a difference for same (992 msec) versus change conditions, t(27) = −2.81, p = .009, demonstrating repetition priming for object shape+colour representations. (As would be expected there was also a difference for new vs. same conditions, t(27) = −4.22, p<.001.) There was also a main effect of colour, with shorter RTs for correctly than incorrectly coloured objects (1004 ms vs. 1095 ms, respectively), F(1,27) = 6.68, p = .015, ηp2 = .20.

For accuracy, the average error rate was 9.8% and there was no evidence of speed-accuracy trade off. There was a main effect of colour, F(1,27) = 9.20, p = .005, ηp2 = .25, with greater accuracy for correctly than incorrectly coloured objects (93% vs. 87%, respectively). There was also a colour x transformation interaction, F(2,54) = 10.01, p<.001, ηp2 = .27. Pairwise t-tests with Bonferroni correction were conducted. For correctly coloured objects there was a difference for same (95%) versus change (89%) conditions, t(27) = 3.38, p = .002, with greater accuracy for objects in the same shape and colour as at pre-test whereas for incorrectly coloured objects there was a difference between same (85%) versus change (90%) conditions, t(27) = −2.46, p = .021, with greater accuracy for objects that changed colour as compared with pre-test.

### Analysis of shape repetition (new vs. change) and shape+colour repetition (same vs. change)

The aim of these analyses was to determine whether there were distinct temporal markers in the ERP correlated with (a) the priming of object shape, and (b) the priming of object shape+colour encoded during the pre-test phase. The rationale was that if the retrieval of object shape and shape+colour memory was associated with functionally and temporally distinct underlying processes then it should be possible to observe unique temporal signatures associated with the localiser contrast for the retrieval of shape independently of colour, and with the retrieval of shape+colour representations. Our main focus was on early ERP waves P1, N1 and P2. Nevertheless, we also examined the P3 wave in order to see whether similar activity occurred at a later stage in processing.

**Figure 1:**
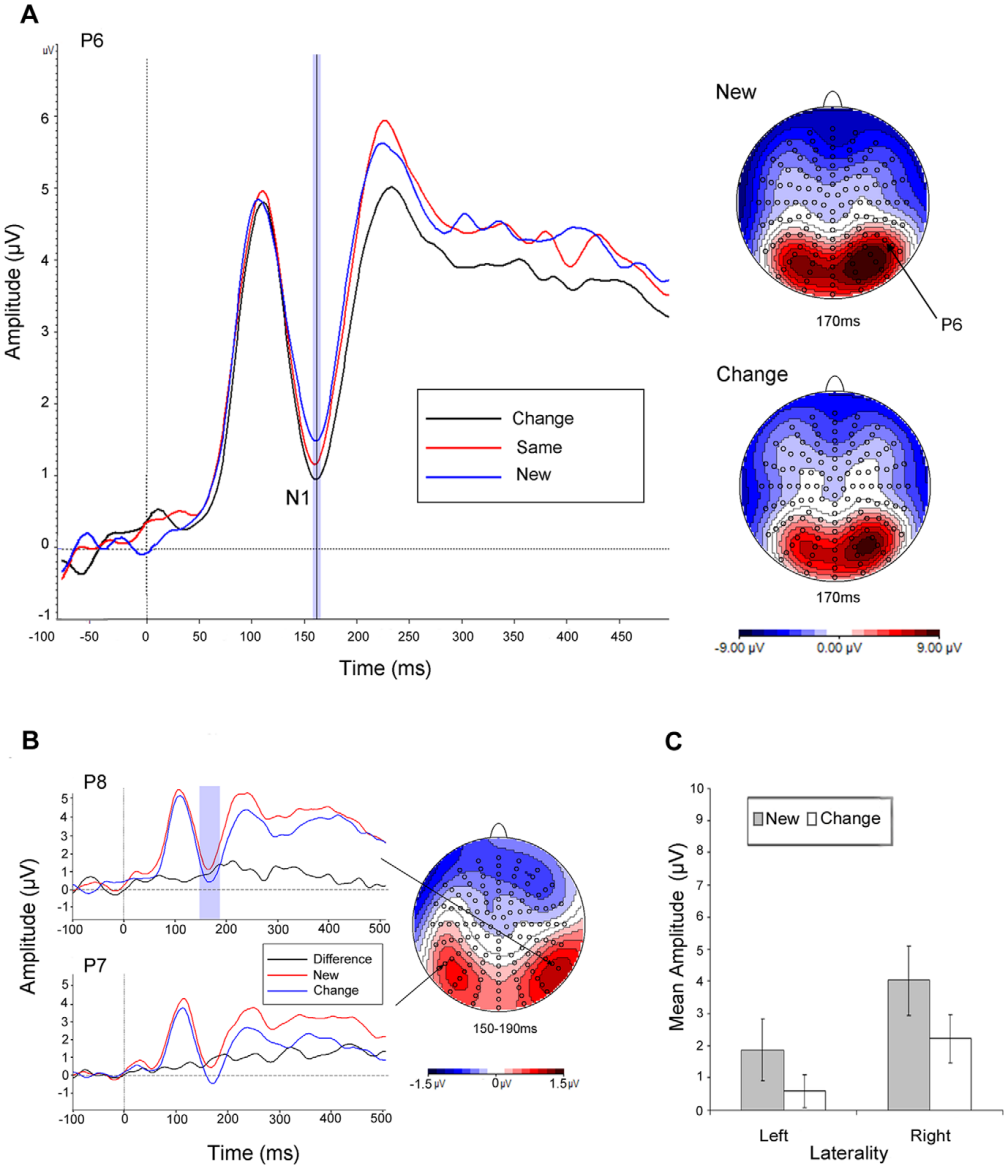
ERP Shape repetition effects. (a) Grand average ERPs to new (blue line) versus change (black line) stimuli plotted between −100 and 500 msec, with respective topographies for the N1 at peak amplitude; (b) topography associated with the difference in mean amplitude for the new versus change contrast over the highlighted 40 msec epoch for the N1; (c) mean amplitudes across posterior electrode clusters during the N1 as a function of laterality and transformation.

**Figure 2:**
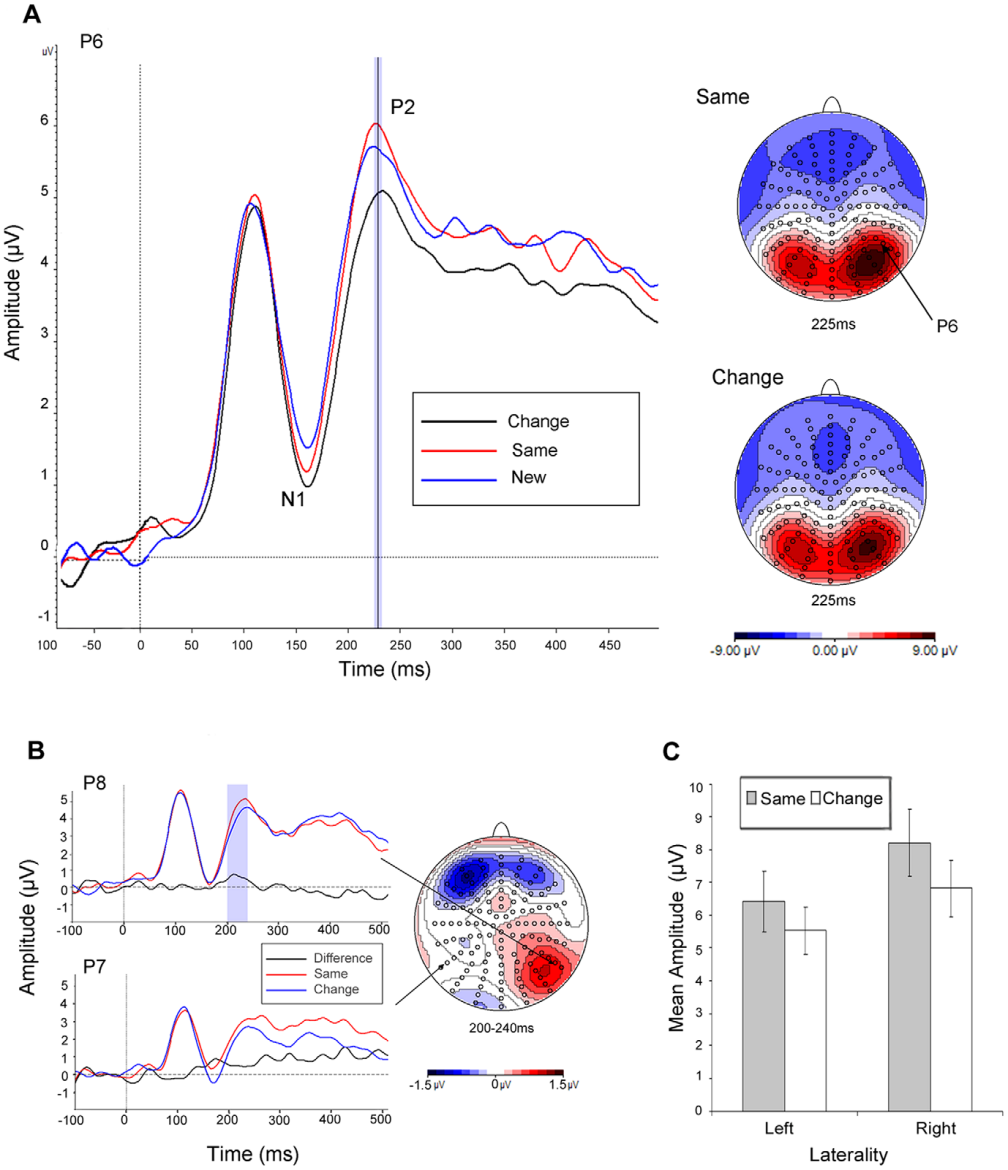
ERP Shape+colour repetition effects. (a) Grand average ERPs to same (red line) versus change (black line) stimuli plotted between −100 and 500 msec, with respective topographies for the P2 at peak amplitude; (b) topography associated with the difference in mean amplitude for the same versus change contrast over the highlighted 40 msec epoch for the P2; (c) mean amplitudes across posterior electrode clusters during the P2 as a function of laterality and transformation.

We first present analyses including all three transformation conditions (*new vs. change vs. same*) for each ERP wave. However, as described earlier the main analysis of interest was based on a region-of-interest approach in which the earliest temporal marker for sensitivity to shape repetition served to define, a priori, electrode clusters over which effects of shape+colour repetition could be analysed subsequently. The shape-repetition marker was identified using the mean amplitude difference wave topography for the *new* versus *change* contrast (see above). This approach allowed us to contrast shape (*new vs. change*) and shape+colour (*same vs. change*) repetition effects over the same electrode clusters. Separate analyses of the ERP waves were undertaken across symmetrical posterior electrode clusters associated with the peak differences in the left and right hemisphere surface scalp potentials. Note, we included laterality (LH/RH) and electrode (1–7) in the statistical analyses, however for clarity and efficiency we only report these factors when they interacted with the transformation conditions (all data are available upon request and the reviewers have seen the full analyses). Mean amplitudes of the P1, N1, P2 and P3 for the transformation conditions are shown in Figures 1 and 2.

#### New vs. Change vs. Same. P1

An analysis of mean amplitude for the P1 (90–130 msec) wave was conducted in order to determine whether attention or low-level perceptual factors may contribute to subsequent differences in the ERP between the transformation conditions (i.e., new vs. change vs. same, collapsed across colour) in the present task. There were no differences in amplitude between the transformation conditions.

#### N1

There was a main effect of transformation, F(2,54) = 7.56, MSe = 94.90, p = .005, ηp2 = .22 (ε = .71) and a transformation x electrode, F(12,324) = 2.82, MSe = 3.60, p = .023, ηp2 = .10 (ε = .38) interaction.

#### P2

There was a transformation x laterality, F(2,54) = 4.46, MSe = 20.12, p = .027, ηp2 = .14 (ε = .75) and transformation x laterality x electrode, F(12,324) = 19.65, MSe = 19.46, p<.001, ηp2 = .42 (ε = .44) interaction.

#### P3

There were no differences in amplitude between the transformation conditions.

#### New vs. Change. P1

There was no difference in amplitude between the transformation conditions.

#### N1

There was a main effect of transformation, F(1,27) = 10.59, MSe = 88.33, p = .003, ηp2 = .28 (ε = 1) and a transformation x electrode, F(6,162) = 3.98, MSe = 3.18, p = .013, ηp2 = .13 (ε = .47) interaction.

#### P2

There was a transformation x electrode, F(6,162) = 3.15, MSe = 12.03, p = .027, ηp2 = .10 (ε = .53) and transformation x laterality x electrode, F(6,162) = 14.41, MSe = 9.01, p<.001, ηp2 = .35 (ε = .66) interaction.

#### P3

There was no difference in amplitude between the transformation conditions.

#### Same vs. Change. P1

There was no difference in amplitude between the transformation conditions.

#### N1

There was no difference in amplitude between the transformation conditions.

#### P2

There was a main effect of transformation, F(1,27) = 5.20, MSe = 16.66, p = .031, ηp2 = .16 (ε = 1), a transformation x laterality, F(1,27) = 5.21, MSe = 22.63, p = .031, ηp2 = .16 (ε = 1), and transformation x laterality x electrode, F(6,162) = 29.56, MSe = 16.91, p<.001, ηp2 = .52 (ε = .47) interaction.

#### P3

There was no difference in amplitude between the transformation conditions.

#### Same vs. New. P1

There was no difference in amplitude between the transformation conditions.

#### N1

There was a main effect of transformation, F(1,27) = 5.69, MSe = 87.76, p = .024, ηp2 = .17 (ε = 1), a transformation x electrode, F(6,162) = 3.16, MSe = 2.95, p = .026, ηp2 = .11 (ε = .54), and transformation x laterality x electrode F(6,162) = 3.09, MSe = 5.66, p = .039, ηp2 = .10 (ε = .43) interaction.

#### P2

There was a transformation x laterality, F(1,27) = 5.32, MSe = 15.01, p = .029, ηp2 = .17 (ε = 1) and transformation x laterality x electrode, F(6,162) = 12.98, MSe = 11.16, p<.001, ηp2 = .33 (ε = .65) interaction.

#### P3

There was no difference in amplitude between the transformation conditions.

### Analysis of colour (correct vs. incorrect)

**Figure 3:**
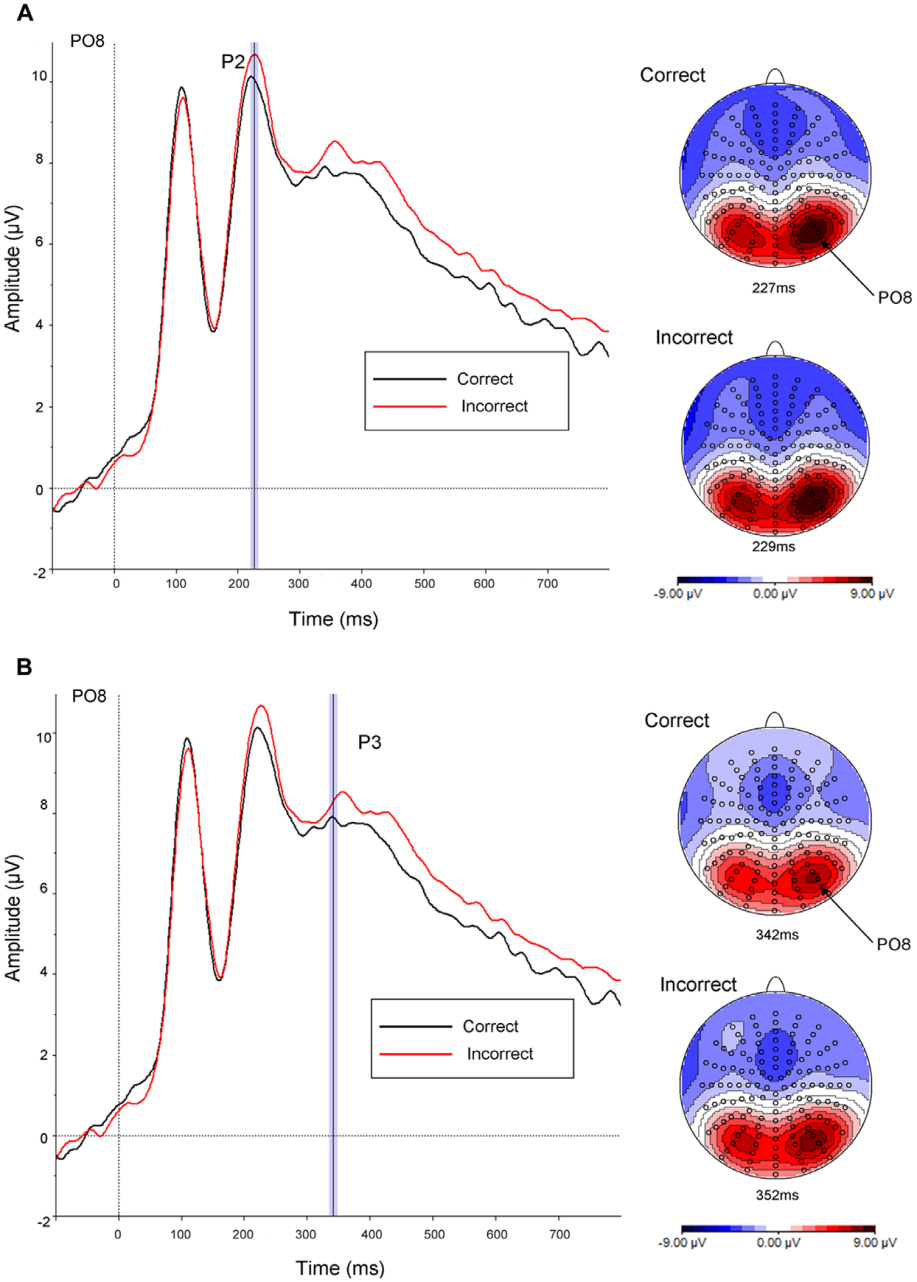
ERP Colour effects. Grand average ERPs to correctly (black line) and incorrectly (red line) coloured objects plotted between −100 and 800 msec with (a) topography associated with the peak amplitude for correctly versus incorrectly coloured objects for the P2; and (b) topography associated with the peak amplitude for correctly versus incorrectly coloured objects for the P3.

The aim of these analyses was to determine whether there were distinct temporal markers in the ERP correlated with object colour by comparing correctly and incorrectly coloured objects presented in the coloured-object decision test. As previously, we only report effects of laterality and electrode when they interacted with the main factor of interest, namely colour. In summary, we found P2 and P3 modulation but not P1 or N1 modulation of mean amplitude associated with the activation of object colour. Mean amplitudes of the P2 and P3 for the colour conditions are shown in Figure 3. There was increased positivity for incorrectly as compared with correctly coloured objects.

#### P1

There was no difference in amplitude between the colour conditions.

#### N1

There was no difference in amplitude between the colour conditions.

#### P2

There was a main effect of colour, F(1,27) = 6.42, MSe = 44.38, p = .017, ηp2 = .19 (ε = 1).

#### P3

There was a main effect of colour, F(1,27) = 10.22, MSe = 55.30, p = .004, ηp2 = .28 (ε = 1).

## Discussion

The main findings were clear cut: (1) we identified a unique early modulation of mean ERP amplitude during the N1 which was associated with the activation of object shape independently of colour. This was indexed by increased negativity for the colour transformed (i.e., change) as compared with the new condition; (2) we also found a subsequent early P2 modulation of mean amplitude over the same electrode clusters associated with the activation of object shape+colour representations. In particular, there was increased positivity for the same as compared with the change condition; (3) these findings were apparent across both familiar and novel shape+colour combinations (i.e., correctly and incorrectly coloured objects); (4) neither of the modulations were evident later in the time course during the P3, in processes associated with the retrieval of semantic knowledge, explicit recollection and decision making [48], [54]; and finally (4) neither of the modulations were likely to be accounted for by attention allocation or low-level perceptual differences between stimuli (such as luminance or contour perception) as evidenced by the absence, in both contrasts, of any modulation of the P1. The P1 is usually thought to be influenced by low level visual parameters such as luminance and contrast, by arousal and by the direction of attention [55]. Indeed, there is evidence that the P1 can be sensitive to attention to colour [56]. The absence of any modulation of the P1 here therefore is consistent with little or no contribution from these factors. Nevertheless, we note that our null finding is suggestive rather than definitive as, for instance, sustained attention can influence both early and late ERP responses [57].

The main aim of this study was to investigate the time course associated with early cognitive processes related to the activation of object shape and shape+colour representations during memory retrieval. We observed perceptual priming of object shape and also object shape+colour early in the time course of processing around 150–200 msec, which was unlikely therefore to be contaminated by explicit recognition (i.e., the parietal P3 wave [58]). For the same reason, we do not believe that the priming observed here reflected changes in associative memory mediated by either stimulus-decision or stimulus-response mappings [59], [60]. For instance, examining word repetition effects, Race et al., [61] found that stimulus-decision and stimulus-response priming were correlated with ERP signatures around the time of response execution whereas like us, stimulus-specific priming was observed early in the time course. Strengthening this view, we required participants to carry out a colour typicality rating task during stimulus encoding whereas during retrieval they made speeded coloured-object decisions. Thus, neither stimulus-decision nor stimulus-response mappings were repeated from study to test (for further evidence against this account of colour priming, see [36]). We conclude therefore that priming reflected the retrieval of perceptual object properties, which is consistent with effects of object colour knowledge on early perceptual processing associated with N1 and P2 during on-line object recognition as reported by Lu et al., [22]. Nevertheless, it would be valuable to examine systematically how object shape+colour binding may operate at different levels of representation and how the different kinds of representation may interact.

We note here that participants' attention in the present experiment was drawn to the typicality of object colour at both study and test through the use of typicality rating and coloured-object decision tasks. This study-test correspondence may have influenced priming, for instance increasing priming as compared with a study task in which participants rated the typicality of the objects as instances of higher order categories such as animal, musical instrument and so on [62]. We selected the tasks to encourage access to colour knowledge, without repeating decision or response processes, in order to accentuate the possibility of observing effects of colour, if present. There appears to be some flexibility, according to processing demands, in the encoding and/or retrieval operations of the implicit representation system(s) for certain properties of objects. For instance, Srinivas [29] found that object priming was influenced by a change in the size of the object when the study and test tasks both required a judgement about the real size of objects. In contrast, there was no influence on priming when study and test tasks required a judgement about the left-right orientation of the object. Flexibility in the inclusion of particular object properties in the memory representation is likely a function of their usefulness in discriminating between different objects; colour in particular is likely to be useful in discriminating between objects that are perceptually similar or perceptually ambiguous and when colour provides a diagnostic cue to identity [63], [64].

Memory for object shape was activated prior to activation of object shape+colour representations. This complements the findings of Proverbio et al. [40], observed in an object detection ERP study. In their study, participants detected a target in a list of coloured objects by attending either to shape or colour. When colour was the relevant property, the N2 was found to be significantly greater when the colour was associated with the object (e.g., *grey elephant*) as compared to when it was not (e.g., *grey strawberry*). However, when shape was the relevant property the N2 was not modulated by colour typicality. Moreover, this was the case even though as an independent feature colour was processed faster than shape. Their study shows that shape can influence colour-based identification of the object. Together the findings from both studies demonstrate that knowledge of object shape and colour are activated early in the time course of object processing but shape is the dominant property which provides access to colour information during on-line identification and memory retrieval. This in turn complements evidence that attention to one particular property of an object can enhance the neural representation of not only that property but also other properties of the same object as compared with those of other objects [65]. It is also consistent with recent models of object recognition in which object representations consist of multi-dimensional structures whereby individual shape elements (e.g., surfaces) are uniquely bound to co-occurring properties such as colour and texture at a different subsequent level of representation [42], [43].

We were also interested to know whether the implicit memorial retrieval of object shape+colour was supported by pre-existing long-term object representations or by new, temporary representations constructed during the course of the experiment. In our paradigm, although there are likely to be pre-existing representations for familiar shape+colour combinations (e.g., *yellow-banana*) that may support priming, it is unlikely that there are pre-existing representations for novel shape+colour combinations (e.g., *blue strawberry*). Therefore, a new memory representation for the novel combination of shape and colour must be constructed and subsequently retrieved in order for priming to benefit performance. We found that the pattern of priming was similar for both familiar and novel shape+colour combinations, nevertheless for the accuracy measure there was a greater probability of successful retrieval for familiar shape+colour combinations. One possibility therefore is that pre-existing (i.e., familiar) representations of object shape supported the retrieval of temporary shape+colour representations.

We may account for our findings in terms of cortical sharpening [32], [33]. The coloured-object decision task required the processing of shape and colour and so when object shape remained the same from study to test but colour was transformed (e.g., from a *yellow* to a *red banana*) a predominantly shape-based representation mediated priming. In contrast, when object shape and colour remained the same from study to test a shape- and colour-based representation mediated priming. One may model these findings using an interactive activation and competition network with bidirectional excitatory and inhibitory connections between object shape and colour [66], [67]. Greater experience with familiar shape+colour combinations would result in stronger mappings between shape and colour and the overall directional strength would be greater from shape to colour than vice versa representing the fact that shape-colour mappings are likely to be one-to-one whereas the colour-shape mappings are likely to be one-to-many. Broadly speaking, for any given object shape there is likely to be a mapping to a single co-occurring colour (e.g., *strawberry* – *red*) whereas for any given colour there are likely to be mappings to many different kinds of object (e.g., *red* – *strawberry*, *traffic light*, *cricket ball*) and it is a general principle of network modelling that one-to-one mappings are more easily learned and processed than one-to-many mappings [68], [69]. Priming may result either from gradual long-term changes to established mappings between shape and colour or temporary ad hoc modifications to the network mediated by attention or working memory (cf., [70], [71]). For instance, as outlined in the Introduction, the priming observed here may reflect inter-trial carry over effects which continuously tune the network, with benefits arising from the preceding trial or trials. This trial-by-trial modulation may be automatic, whereby participants cannot help but be influenced by information processed on the previous trial(s), or subject to top-down attentional control [14].

We also observed independent effects of colour, as measured by directly comparing correctly and incorrectly coloured objects, and behavioural performance was correlated with modulation of mean ERP amplitude during the P2 and P3. The P2 modulation is consistent with the retrieval of object colour knowledge as described earlier. Concerning the cognitive processes associated with the P3 modulation there are two main possibilities. First, that it reflects a form of semantic conflict arising for incorrectly as compared with correctly coloured objects. A number of studies have shown that the amplitude of the ERPs elicited by incongruent and congruent colour-word stimuli (e.g., the word *blue* written in either green or blue ink) differ in the range of 300–450 msec, although there is no consensus on the topographic distribution of these differences which may be in either central-parietal or centro-frontal locations. Furthermore, some studies have identified the P3 wave, observing that the amplitude elicited by incongruent stimuli is smaller than that elicited by congruent stimuli, and it has been suggested that semantic conflict may delay the start of the response selection process [72], [73]. However, the semantic conflict account runs into some difficulty here as the amplitude elicited by incorrectly coloured stimuli was actually larger than for correctly coloured stimuli. A related possibility, more in keeping with the present finding of larger amplitudes for incongruent trials and a posterior topographic distribution, is that the P3 modulation reflects the activation of stored perceptual colour information in order to guide a response when colour and object shape information activate incompatible semantic representations on incongruent trials [74]. Alternatively, it may be that the P3 modulation reflects a categorization process whereby more novel or deviant items (i.e., incorrectly coloured objects) produce larger P3 amplitudes during the process of being classified as members of a novel set [75].

Finally, we note that it would be of interest to extend the pattern of findings presented here to different categories of object, for instance by comparing natural versus man-made objects. There is evidence that early in development children use object shape to form categories and learn new words [76], [77]. In a phenomenon known as ‘shape bias’ children generalize a novel word to objects that are similar in shape rather than colour, size or texture [78]. Moreover, this bias is influenced by differences in perceptual similarity and complexity amongst objects, and hence likely also object category [79], [80]. Of further interest, in the neuropsychological literature there is evidence of category-specific selective deficits in object processing, with dissociations observed between animal, fruit and vegetable, and man-made categories (for a recent review, see [81]). The present design was not optimal for exploring this issue as (a) there are likely to be a number of important early attentional and perceptual differences between object categories, for instance in terms of within-category similarity and complexity, that were not controlled; (b) such differences make it difficult to interpret ERP modulations later on in the time course; and (c) there were a limited number of objects and so manipulating category would reduce power. Nevertheless, we carried out a post hoc examination of the data contrasting natural and man-made objects. As expected, there were P1 and N1 wave modulations with increased P1 positivity and decreased N1 negativity for natural objects, which are difficult to interpret. Perhaps of more interest, there was also an early P2 modulation with increased positivity for natural objects. However, this P2 effect was independent of any effects of object transformation. One tentative possibility is that the category difference for the P2 wave arose because object shape and colour information was more strongly bound together for natural objects. Nevertheless, we note that it is also the case that many natural objects change colour with ripening and decay (in particular, fruit and vegetables) and in their case colour may be less strongly bound to shape than for man-made objects.

In conclusion, we have provided the first evidence on the timing of activation of perceptual object shape and shape+colour representations during implicit retrieval. We identified distinct ERP waves indexing early perceptual processes, namely N1 and P2, associated with the initial retrieval of shape and the subsequent retrieval of both familiar and novel shape+colour representations formed after just a single encounter with the object. Importantly, ERPs varying with repetition priming of object shape and colour reveal the cortical processes of knowledge retrieval and provide markers for future work on object representation and memory.

## Materials and Methods

### Ethics statement

The study was approved by the Research Ethics Committee of the Department of Psychology, Swansea University and Bangor University. All participants gave written informed consent and were treated in accordance with the principles expressed in the Declaration of Helsinki.

### Participants

Twenty-eight participants recruited from Swansea and Bangor universities (mean age = 29.9, standard deviation [s] = 6.9, 17 females, all right handed) took part in return for a small payment.

### Stimuli

**Figure 4:**
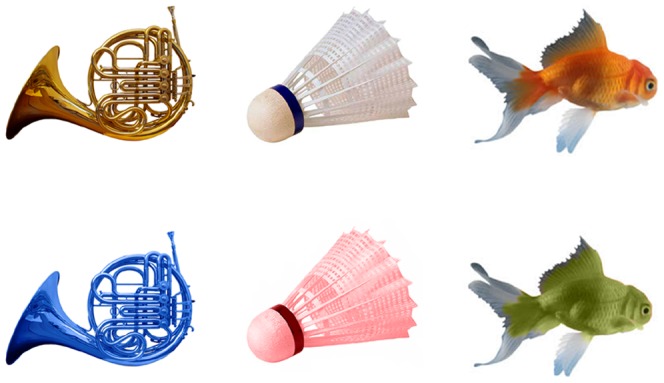
Examples of correctly (top) and incorrectly coloured objects.

The stimuli consisted of one hundred and fifty pictures of objects encountered in the real world. Most pictures were taken from a photographic website (November-December, 2006, www.photoobjects.net) with a subset selected via an internet image search using the Google search engine. The objects were coloured either as typically encountered in the natural environment (correct colour conditions) or were matched on all image characteristics (e.g., complexity, spatial frequency composition, scale, and compactness) but their colour was incorrect (incorrect colour conditions). The stimuli were selected on the basis that each object had an associated surface colour where the surface colour of each object was based on colour agreement scores obtained by Joseph [82] and Vernon and Lloyd-Jones [36]. Seventy-five objects were taken from Lloyd-Jones and Nakabayashi [83]. Participant agreement on object colour was assessed in the same way as Lloyd-Jones and Nakabayashi (p.313) using colour perception and colour memory conditions, with an independent group of 16 participants. In the *perception* condition participants indicated the surface colour of the object and in the *memory* condition they were given the names of the objects and assigned the most prototypical colour. Average participant agreement on object colour was 78%. We used the imaging software Adobe Photoshop CS2 to create an incorrectly coloured version of each correctly coloured object. To do so, we rotated the correct colours across objects whilst ensuring that correctly and incorrectly coloured objects were matched for colour frequency and luminance. Thus, an incorrectly coloured object was created by selecting the surface colour of a correctly coloured object and pasting the colour onto a different object using the colour replacement tool. The lightness of the colour-replaced object was adjusted using the brightness contrast tool. We then measured the luminance of the stimuli directly from the computer monitor using a Minolta L-100 luminance meter. The luminance values for correctly and incorrectly coloured objects were 58.4 cd/m^2^ (s = 16.2) and 59.2 cd/m^2^ (s = 16.6) respectively, and there was no difference between correctly and incorrectly coloured objects (t(149) = −.94, p = .35). The stimuli were selected and presented using a rotation design to ensure an equal distribution of all stimuli across all the conditions. On average the stimuli subtended 2.2° horizontally and 2.5° vertically, for both correctly and incorrectly coloured objects. Example stimuli are shown in Figure 4.

### Design and Procedure

The study consisted of two phases: pre-test (colour typicality rating) and test (coloured-object decision). In the pre-test phase participants were presented with 100 stimuli and were required to rate object-colour typicality on a scale from 0 (‘not typical at all’) to 7 (‘highly typical’) using a standard computer keyboard. The data from this part of the session was not analyzed as the purpose of the pre-test phase was to allow participants sufficient time to memorize the stimuli without forewarning them that they would be tested on the material subsequently. Nevertheless, we checked the data to confirm that all participants were performing the rating task correctly. During the test phase 150 stimuli were presented. The participants were required to complete a speeded coloured-object decision task in which they classified the colour of each of the presented stimuli as ‘correct’ or ‘incorrect’ for the object and the binary choice was expressed by using numerical keys 3 on the number lock keyboard and 4 on the main keyboard. The response keys were counterbalanced to correct for handedness.

Transformation in object colour between the pre-test and test phases resulted in a 2 (colour – correct/incorrect)×3 (transformation – same/change/new) within-participants design for the test phase. Thus, half the objects in the test phase were correctly coloured and half were incorrectly coloured. Within each of these colour conditions, stimuli could be the same as at study (i.e., same correct or incorrect colour, the *same* condition), in a changed colour from study to test (i.e., correct colour at study and incorrect colour at test or vice versa; the *change* condition) or new stimuli (half correctly coloured and half incorrectly coloured) which served as baselines against which to measure priming. (Note, in this way changes in colour alone occurred during the experiment; there were no combined changes in both shape and colour.) There were therefore 6 test conditions in all, each with 25 items rotated through the pre-test and test phases ensuring that each stimulus appeared equally often in each of the phases across the experiment.

The procedure was as follows. At pre-test, stimuli were presented sequentially for 5 secs each following a 250 msec fixation cross. Participants rated the typicality of the colour for the associated object and made a response once the stimulus had disappeared from the computer screen. The pre-test phase was then followed immediately by the test phase where each stimulus was presented following a 250 msec fixation cross and a 500 msec blank screen. The stimulus remained on the computer screen until a response has been made. Participants engaged in speeded key press responses to indicate whether the object was correctly or incorrectly coloured.

### Electrophysiological recording and processing

The electroencephalograph (EEG) was recorded continuously through 128 electrodes placed on an ECI cap (Electro-Cap International, Ohio, USA) using the Active-Two Biosemi EEG system (Biosemi V.O.F Amsterdam, Netherlands). Eye movements and blinks were corrected using the ICA protocol in Analyser 2 software and segmented data was then visually inspected with trials containing artefacts rejected. Epochs that contained muscle or skin potential artefacts were rejected. Only trials on which participants gave a correct response were included and the mean number of correct trials after artefact rejection was: 41.5 (same), 41.7 (change) and 41.6 (new). Activity from all electrodes was sampled at a rate of 512 Hz. Offline 30 Hz (48 db/oct slope) lowpass and .1 Hz (48 db/oct slope) highpass filters were applied to the data. All data was re-referenced to an average reference which was then used to generate the grand averages. We used a 100 msec pre-stimulus interval for the baseline correction. Continuous recording took place during the test phase of the experiment and trials were epoched/segmented from 100 msec pre-stimulus to 800 msec post-stimulus onset.

### EEG analyses

The three main early ERP waves P1, N1, P2 and the later P3 wave were identified based on the topography, deflection and latency characteristics of the respective grand average ERPs time-locked to stimulus presentation. Preliminary epochs of interest for each wave were defined on the basis of deflection extrema in the mean global field power (MGFP) [84]. Peak detection was time-locked to the electrode of maximal amplitude for each wave. The latency of peak amplitude was used to define equal time epochs of 40 msec for analyses of the three early waves: P1 (90–130 msec; Peak latency (B7) = 110 msec); N1 (150–190 msec; Peak latency (D24) = 170 msec); P2 (210–250 msec; Peak latency (B7) = 230 msec); P3 (300–380 msec; Peak latency (B7) = 340 msec).

The topography associated with differences in mean amplitude for the pairwise contrast of the *new* versus *change* conditions was computed by subtracting the respective grand average amplitudes between conditions for each electrode (see below). This difference wave topography was used to identify, a priori, spatially contiguous electrode clusters showing the earliest statistically reliable sensitivity to shape repetition in the event-related potential. Thus, it served as a temporal localiser contrast to define an index or temporal marker for shape-related processing. Using this method, two symmetrical clusters over the left (LH) and right (RH) hemispheres were extracted each consisting of seven spatially adjacent posterior electrodes: RH: B5, B6, B7, B8, B10, B11, B12 and LH: A8, A9, A10, A11, D29, D30, D31 which correspond/overlap with electrode locations P4, P6, P8, PO8 and P3, P5, P7, PO7 of the extended 10–20 system respectively. These electrode clusters formed the region-of-interest for the subsequent analysis of shape+colour repetition, namely *same* versus *change* conditions. Mean amplitudes were analysed using the General Linear Model by way of repeated measures ANOVA. Greenhouse-Geisser corrections were applied to all analyses of ERP data [85]. Note, the topographic views depicted in the figures present all electrode locations viewed at 120 degree maximum angle.
